# Molecular Interrogation of the Feeding Behaviour of Field Captured Individual Insects for Interpretation of Multiple Host Plant Use

**DOI:** 10.1371/journal.pone.0044435

**Published:** 2012-09-19

**Authors:** James P. Hereward, Gimme H. Walter

**Affiliations:** 1 School of Biological Sciences, The University of Queensland, Brisbane, Queensland, Australia; 2 Cotton Catchment Communities Cooperative Research Centre, Australian Cotton Research Institute, Narrabri, New South Wales, Australia; University of Miami, United States of America

## Abstract

The way in which herbivorous insect individuals use multiple host species is difficult to quantify under field conditions, but critical to understanding the evolutionary processes underpinning insect–host plant relationships. In this study we developed a novel approach to understanding the host plant interactions of the green mirid, *Creontiades dilutus*, a highly motile heteropteran bug that has been associated with many plant species. We combine quantified sampling of the insect across its various host plant species within particular sites and a molecular comparison between the insects' gut contents and available host plants. This approach allows inferences to be made as to the plants fed upon by individual insects in the field. Quantified sampling shows that this “generalist” species is consistently more abundant on two species in the genus *Cullen* (Fabaceae), its primary host species, than on any other of its numerous listed hosts. The chloroplast intergenic sequences reveal that *C. dilutus* frequently feeds on plants additional to the one from which it was collected, even when individuals were sampled from the primary host species. These data may be reconciled by viewing multiple host use in this species as an adaptation to survive spatiotemporally ephemeral habitats. The methodological framework developed here provides a basis from which new insights into the feeding behaviour and host plant relationships of herbivorous insects can be derived, which will benefit not only ecological interpretation but also our understanding of the evolution of these relationships.

## Introduction

A clear understanding of the behaviour of individual insects is crucial to interpreting many ecological and evolutionary phenomena, for it informs about the extent and limits of variation within a population (or species) and about differences between populations or species. Ascertaining the feeding behaviour of herbivorous insect individuals under natural conditions is difficult, especially in those species that use multiple hosts, but it is crucial to defining host-plant interactions accurately. Although laboratory studies of host plant use do provide insight into how individuals use host plants of alternative species, they suffer several compounding limitations, including the difficulty of incorporating and testing long range host searching mechanisms, the exclusion of environmental influences, and the difficulty of reconciling behaviour observed in the laboratory with that observed in the field [1]. To determine what individuals feed on in the field requires not only observations of an insect on a host plant, but often a method of testing the feeding history of that individual relative to alternative host plants in the area. In this paper we elucidate the feeding behaviour of individual green mirids (*Creontiades dilutus*), a species of bug recorded from multiple host plants, under natural conditions. This required that a methodological approach be developed, based on a combination of structured sampling in the field and gut content analysis, as expanded below.

The use of multiple host plant species by an insect herbivore is usually determined through the scrutiny of host plant lists, but these comprise, at best, summary statements. Many such records are simply incidence records. The observed occurrence of an insect on a host plant does not necessarily confirm regular feeding or reproduction on that plant. This shortcoming can be overcome to some extent by using the presence of juveniles as an indication that a host is significant to the life cycle of that insect species. However, for species with highly motile juvenile stages (such as lepidopteran caterpillars and many orthopteran and hemipteran species) it can be difficult to be sure that juvenile presence on a plant truly represents feeding on that host. Furthermore, the relative importance of the host plant species to the ecology of the herbivore in question may be distorted by such incidence records, and their summary into host plant lists [2].

In this study we interrogate the feeding behaviour of green mirid individuals under field conditions. Not only is this species usually characterised as a “generalist” on the basis of both adults and juveniles commonly being found on many host plant species [3]–[6], but it is also highly motile. Sampling of crops to establish patterns of invasion into cotton [4], and microsatellite based analyses of migration (JPH Unpublished data) indicate that these insects move long distances (at a scale of at least 2000 km) between the arid interior of Australia and eastern cropping regions. They are also highly motile within a locality (both adults and juveniles), fleeing in response to any disturbance (pers. obs. JPH). Although this particular mirid species is endemic to Australia [7], and was likely restricted to the arid interior prior to European settlement when land was cleared to establish broad scale agriculture, it has been recorded from only a few native Australian host plants (based on those incidence records that are available). An initial survey in this region did, however, implicate the native leguminous forb *Cullen cinereum* as a major host [4], based on relatively high numbers on this plant. Thus, the use of multiple hosts by green mirid individuals in inland Australia, in particular, warranted further investigation.

Whereas mirids are often regarded as ‘sap sucking bugs’, they do not feed on phloem or xylem, as many hemipterans do. Instead, phytophagous mirids like *C. dilutus* use their stylets and watery saliva to lacerate and macerate a pocket of cells [8]. *Creontiades dilutus* saliva has a complex mix of proteases, and pre-oral digestion of plant tissue is evidently an important aspect of their feeding [9]. The resultant mix of semi-digested plant cells and tissue is then consumed, which makes it probable that chloroplasts are also consumed by *C. dilutus*.

A few studies have used chloroplast sequences to recover the gut contents of herbivorous insects, and thus determine directly which host plant species have been fed upon. To this end, chloroplast markers have been amplified from DNA obtained from dry coleopteran material in museum collections [10], and also from wild caught beetles [11]. These studies could not, however, relate dietary information directly to putative host plants because they relied on publically held database records of chloroplast sequences. The taxonomic resolution of host plants has thus depended on the somewhat limited taxonomic coverage of records in these databases.

Through quantitative sampling of mirids on known host plants as well as potential host plants growing together across different localities, we were able to quantify the relative importance of each host species. We collected tissue samples from the range of plant species from which *C. dilutus* had been collected in each locality. These plants were identified and DNA extracted from both the plants and also the mirids collected from them. We then amplified chloroplast intergenic sequences from the plants and from individual insects to provide a direct link between insect individuals and the plants on which each had fed (within about 48 hr prior to capture).

We show how this combination of ecological sampling data and molecular diet analysis provides new insights in understanding the ecology of insect feeding behaviour and for interpreting their host plant relationships in the field. Use of the proposed methodological and conceptual framework will therefore develop broader understanding of the ecological and evolutionary significance of the use of multiple plant species by herbivorous insects.

## Results

**Figure 1:**
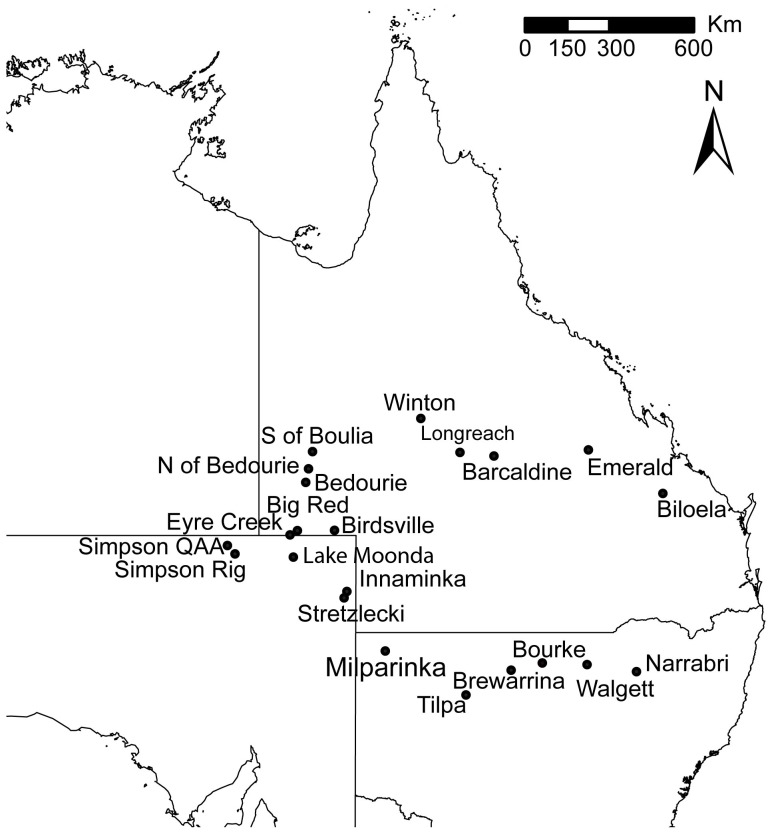
Map of northeastern Australia showing the sampling locations for the field survey.

**Figure 2:**
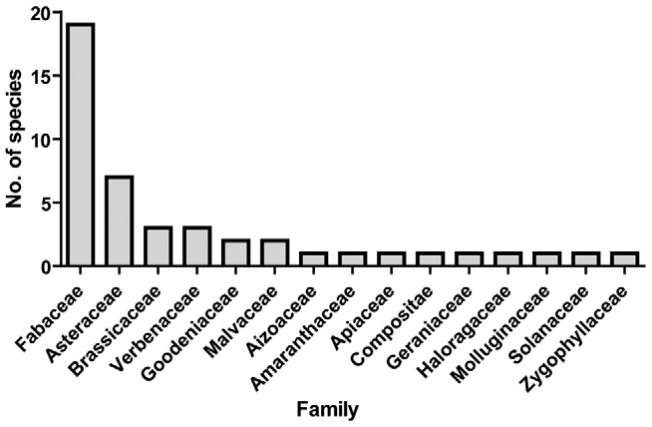
Number of host plant species per family for which records exist of *Creontiades dilutus* nymphal presence. Data from the survey reported in this paper and from records in the literature (see Methods and Table S1 for details).

The extensive host plant survey in this study (Fig. 1, Table S2) revealed 26 new putative host species, 22 of which are listed as Australian native species (Australian Virtual Herbarium http://www.ersa.edu.au/avh/; Table S1). When combined with existing records, a total of 97 potential host plant species has now been recorded for *C. dilutus* (Table S1). When hosts that have no record of *C. dilutus* nymphal presence are removed (54% of the total), this list is reduced to 45 host plant species across 15 families, but primarily Fabaceae (42% of those host species with nymphs recorded) and to some extent Asteraceae, with 16% (Fig. 2).

The field survey of *C. dilutus* host associations and abundance covered a circular transect of 6000 km through central Queensland, the southeast corner of the Northern Territory, and northern New South Wales. The area was selected because green mirids had been collected there before, it supports the ephemeral vegetation that typifies green mirid hosts, and other insects associated with such plants are known to invade sub coastal agriculture from there [12]. As is typical of these arid regions [13] rainfall was temporally and spatially patchy during the season of this sampling. Suitable host plants (forbs and herbs) generally require more than one rainfall event (and this is usually highly localised) to flourish, adding to their patchy occurrence. Such localities are typically interspersed with large areas (often several hundred kilometers) of barren land.

Our quantitative sampling at the 22 sites where *C. dilutus* was present (of 82 likely sites investigated) revealed that the five plants on which green mirids were most numerous are all in the genus *Cullen* (Table S2), and the highest number of mirids collected from a *Cullen* host (344 total, 5 m^2^ sweep-net samples, n = 10) was over 4 times higher than the highest number retrieved from a non-*Cullen* host (*Crotalaria eremaea*, 80). However, not all *Cullen* species hosted large numbers of these bugs, as site-specific factors such as temperature extremes and time since colonisation also affect insect abundance. As with available potential hosts, *C. dilutus* was patchily distributed across the inland sites sampled, but most abundant where *Cullen* plants occurred (Table S2). This could indicate that the presence of green mirids on adjacent plants may be spill-over from *Cullen* hosts. We therefore assessed the abundance of *C. dilutus* on a site by site basis, for those sites where *C. dilutus* had been sampled from *Cullen* host plants as well as other plant species.

**Figure 3:**
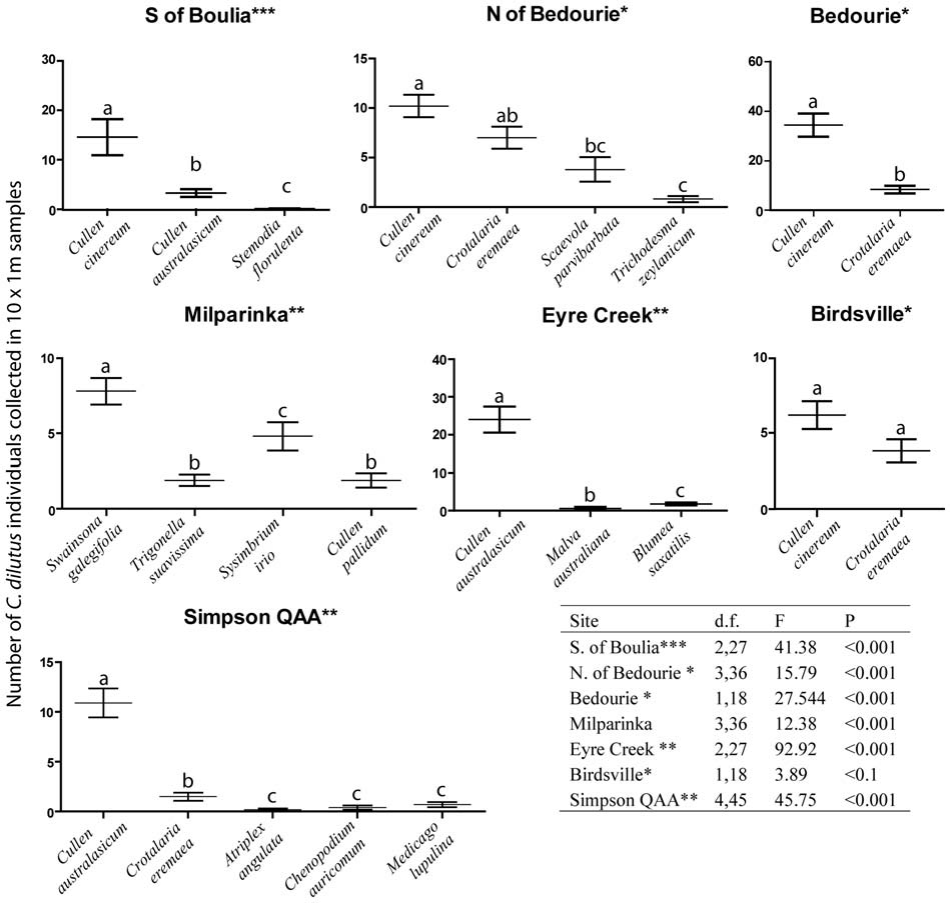
Abundance of *C. dilutus* across host plants at seven sites in northeastern Australia where this bug was located and both *Cullen* and alternate host plants grew together (bars represent the mean and the error bars are +/− 1SE, n = 10). For a given site, bars with the same letter above them are not significantly different from one another, per ANOVA and Fisher's LSD test with a Benjamini-Hochberg correction for multiple pair wise comparisons. * no transformation required, **log transformation applied, *** log(log) transformation applied.

*Creontiades dilutus* abundance was statistically different across potential host plant species at six of these seven sites, with only Birdsville returning no significant difference at P<0.05 (Fig. 3). Abundance was consistently higher on *Cu. australasicum* and *Cu. cinereum* than on alternative hosts (Fig. 3). However, the third *Cullen* species sampled, *Cu. pallidum*, at Milparinka, had a significantly lower abundance of *C. dilutus* (mean 1.9+/−0.48) in comparison to the two host plants with the highest abundance there (*Swainsona galegifolia* 7.8+/−0.89, *Sysimbrium irio* 4.8+/−0.92) at that site.

In *C. dilutus*, the chloroplast *trnL* intron used by [10] did not amplify with a high success rate, probably as a result of degradation by extra-oral digestion in these bugs, which is likely to reduce the number of larger DNA fragments remaining in the insects gut. We therefore selected the *trnL-trnF* intergenic spacer which is generally a smaller region (158–438 bp as opposed to 389–614 bp) [14] and therefore more amenable to PCR amplification from degraded DNA. The *trnL-trnF* intergenic spacer amplified in 100% of our host plant DNA samples (21 species in 8 families) amplifying fragments from 161 to 567 bp. These sequences were highly variable across families with many insertions and deletions and a single alignment could not be produced to assess sequence divergence. Five separate alignments were produced that correspond to the 5 families for which we had sequenced more than one species, leaving three sequences un-aligned. With the exception of the three species of *Cullen* (which only differed from one another by one bp substitution) the closest sequences in our data were those of *Senecio gregorii* and *S. depressicola* which had 2.1% base difference, for all other species it was considerably higher. We therefore set a threshold of 2% difference to define a *trnL-trnF* match for the insect derived fragments, but this value is arbitrary and a match should not be considered a robust plant species identification.

The amplification success rate of the chloroplast marker in insect-derived DNA was relatively low (28.5%, 288 insect samples), yielding 82 good sequences (length = 80–398 bp after poor quality sequence was removed). This likely represents a limitation of gut content analysis in mirids by means of PCR, because their extra-oral digestion probably degrades DNA. The size variation in this fragment was such that it allowed more than one sequence to be recovered from each of 5 insect samples by agarose gel recovery representing feeding on more than one host plant. These five included two of the individuals from Eyre Creek (Table 1) one returned both *Cullen* and *Sysimbrium irio* fragments and one returned both *Cullen* and *Chenopodium auricomum* fragments. The other 3 samples for which multiple feeding was detected were from sites that had low numbers of mirids sequenced, and were not included in the analysis presented here. Specifically; one individual from Simpson desert that had both *Crotalaria eremea* and *Cullen australasicum* fragments, one individual from Simpson that had *Senecio gregorii* and *Blennodia pterosperma* fragments, and one from Milparinka that had both *Cullen* and *Phlegmatospermum cochlearinum*. In addition, 4 of the sequences were of poor quality and probably also represented feeding on multiple hosts. These four sequences were not recovered by cloning, and instead were discarded from the analysis. It is also possible that closely related plants that were not sampled in our plant dataset may not have been diagnosed with the *trnL-trnF* fragment used (as for the *Cullen* sequences, see below). Our results are therefore conservative in underestimating the use of multiple hosts by individuals of this species.

**Table 1:** Gut-derived chloroplast sequences from the green mirid *Creontiades dilutus* showing the number of sequences that match the host (N host) from which the insects were sampled, and the number that match a plant other than the one from which the insects were sampled (N different).

Host plant sampled	Site	N host	N different	Species recovered
*Blumea saxatilis*	Birdsville	9	1	*Panicum*
*Cullen australasicum*	Simpson QAA	8	1	*Chenopodium auricomum*
*Calotis plumulifera*	Simpson QAA	9	1	*Cullen australasicum*
*Cullen australasicum*	Eyre Creek	6	3	*Chenopodium auricomum*
				*Calotis plumulifera*
				*Sisymbrium irio*
*Brachysome campylocarpa*	Lake Moonda	11	0	
*Senecio gregorii*	Stretzlecki	17	0	

Plant species on which *C. dilutus* had fed but was not collected from are listed (Species recovered).

The fragment amplified from both the plants and the mirids collected from these plants was diagnostic for all plant species that we had sequenced using a threshold of 2% difference, with the exception of the three species of *Cullen*. The sequences from these three host species differed by only one site toward the *trnF* end of the plant sequences and this site was absent from many of the *Cullen* sequences obtained from insect DNA. More than one species of *Cullen* was, however, never present at the same site, so the gut derived sequences could be assigned to host species based on the availability of that host at any given site. There were only two instances where the host plant detected in the mirid was not in our set of plant-amplified chloroplast sequences, in the first instance (C2721, Genbank accession JX134164) a Genbank search indicated that this might be *Sysimbrium irio*, pair wise alignment with this sequence (Genbank accession DQ180275.1) gave 1.4% difference and we defined this as a match although the 2% threshold used is arbitrary and this identification should be considered provisional. In the second (C1501, JX134132) *Panicum virgatum* was the closest sequence available on Genbank (e-value = 2E-116). When our sequence was pair wise aligned to the *P. virgatum* complete chloroplast genome (Genbank accession HQ731441.1) there was 4.2% difference, which is outside of our 2% threshold, and we assigned this sequence to the genus *Panicum*.

Host plant collections for which less than 5 sequences had amplified successfully were excluded from this analysis leaving 66 insect-derived chloroplast sequences (Genbank accessions JX134132–JX134197). Of these 66 sequences, 10% showed that the green mirid individuals had fed on a plant other than the one that they had been collected from. Even when collected from *Cullen* hosts a high proportion of individuals had fed on a different plant species (Table 1).

## Discussion

To explore the host plant relationships of this highly motile insect, with a broad reported host range, we developed a framework that integrates quantified spatial host plant sampling with molecular analyses of recent plant food intake. This framework goes beyond incidence records, allowing inference into the rates of host plant species use and recent feeding behaviour of individual insects. The ability to make this inference for field collected insects means that a critical assessment of the relationship between an insect and multiple hosts can be made without the limitations of laboratory studies. We discuss the findings of this approach specifically in relation to *C. dilutus*, then consider the implications of our results and approach more broadly.

### Host plant relationships of C. dilutus

*Creontiades dilutus* is highly motile and is endemic to Australia [7]. A large number of incidence records demonstrate that these bugs feed on multiple hosts. Green mirids were likely restricted to the arid interior of Australia prior to European settlement and the spread of agriculture. This implies, in turn, that the species has close evolutionary relationships to plants in this area (see introduction). Before this study the host plant relationships of *C. dilutus*, in particular outside of agricultural areas, was not fully resolved. Our aim, therefore, was to investigate the use of multiple hosts by this species, particularly in central Australia.

Our data do confirm that *C. dilutus* uses many host plant species, most of which are in the family Fabaceae (Fig. 2). However, the abundance of *C. dilutus* is consistently higher on plants in the genus *Cullen* than on other host plant species surveyed. Specifically, the Australian native species *Cu. cinereum* and *Cu. australasicum* are identified as primary hosts for green mirids by the quantitative host plant sampling presented here. Not only is the highest mirid abundance recorded on these species, but on a site by site basis these two *Cullen* host plants have significantly higher abundance of *C. dilutus*, across six sites, compared to other plant species sampled locally (Fig. 3). *Cullen australasicum* and *Cu. cinereum* are morphologically similar to one another, but *Cu. pallidum* is densely covered in hairs, which may explain why this latter species seems to be a relatively poor host for green mirids (Fig. 3). Alternatively, the chemical cues used by *C. dilutus* for host location and feeding initiation may well differ across these *Cullen* species, but this requires further investigation.

Simultaneous sampling of the insect and the host plants available locally allowed a molecular comparison of the insect gut contents (at the time of sampling) with the host plant from which it was collected. This underpins an inference of feeding behaviour beyond just incidence of the insect on a plant. Our molecular analysis of host plant feeding in *C. dilutus* shows that this species often feeds on host plant species other than the one from which it had been collected, even when they were collected from their primary host (Table 1). The behaviour that this represents is particularly striking considering that fragments of the length that we amplify here can evidently be detected for only as long as 12 to 48 hrs post ingestion [15]–[17].

The behavioural implications for the mirids appear somewhat contradictory, however. Whereas green mirid abundance is much higher on *Cu. australasicum* and *Cu. cinereum* than on other host plants nearby, individuals collected from these primary hosts evidently do move between different plant species locally and feed on these other hosts, even species that are relatively insignificant in terms of mirid abundance. The host use of generalist species is often viewed in the context of optimisation strategies [18] and enemy free space [19]. Optimal diet mixing, for example, has been suggested to favour resource generalisation through individual fitness gains. However, feeding trials on *Nezara viridula*, a heteropteran that uses multiple host species in a similar way to *C. dilutus*, show that diet mixing does not provide direct fitness benefits. The use of multiple hosts does, however, allow this species to persist on sub-optimal plant species when their primary host species are not available [20]. In the arid interior of Australia, *C. dilutus* is associated with spatially and temporally patchy resources that are highly dependent on recent rainfall. Except in years of unusual rainfall, precipitation events and plant growth tend to be localised. We suggest that the use of multiple hosts represents a similar behavioural adaptation to that of *N. viridula*, and this allows these bugs to survive and reproduce within a patchy and ephemeral environment.

This study has focussed on the relationships between *C. dilutus* and native host plants in the arid interior of Australia. At the time of sampling (winter) green mirids were present only in very low numbers on agricultural crops sampled; effectively zero in our standardised sampling (Table S2). In the summer, by contrast, green mirids are very difficult to locate in the arid interior, as it is far too hot and dry to support plant growth, but in agricultural regions they reach much higher densities on lucerne (*Medicago sativa*, Fabaceae) than on any other crops [4]. Their densities on lucerne reach almost as high as on the *Cullen* primary hosts (JPH unpublished data 2007–2008), with irrigation in agricultural areas being significant in this respect.

Our confirmation that *Cullen* species are primary hosts, and the revelation of multiple-host feeding over a short time, highlights several questions regarding the higher abundance of *C. dilutus* on these two species relative to other host plants in arid Australia, and its relationship to lucerne where that is cultivated. The specific cues (olfactory or visual) that *C. dilutus* uses to locate hosts and initiate feeding may be shared across *Cu. cinereum*, *Cu. australasicum* and lucerne. Alternatively, green mirids may perform better on these hosts in comparison to other plant species. Targeted research into the host searching behaviour of green mirids and the specific cues to which they respond would begin to answer these questions. Host performance testing is difficult in this species, as it has proved impossible to maintain a laboratory culture for more than three generations; the research presented here indicates, however, that using *Cullen* hosts in the laboratory may be a possible solution to this problem.

### Future use of this framework

Molecular techniques are increasingly being employed to analyse the diet of wild organisms [21]–[23]. In insects such studies have tended to focus on predation, requiring that specific assays are developed [15]–[17], [24], [25]. The use of chloroplast sequences provides a general approach to assessing herbivorous insect diets [10], although it has not been applied to answer specific questions about polyphagous species until now. Some of the most significant agricultural pests are polyphagous insects, and polyphagous habits are difficult to explain in evolutionary terms [26], [27]. The conceptual and methodological framework we propose here provides a targeted approach to interrogating the recent feeding history of individuals under field conditions. It does so by combining the quantified spatial sampling of insect abundance across multiple hosts in the field with a molecular comparison between the gut contents of these samples and the locally available host plants. By contrast, a bar-coding only approach to diet analysis could not have highlighted the contrast between insect abundance across different host plant species and individual behaviour in the same way. The work presented here is a “proof of concept” evaluation of the combined approach. Through it we illustrate how this combination of techniques can illuminate host use in a way that incidence records cannot, for it reveals where insects have actually been feeding in the field. Getting such information in any other way would be intractable without molecular techniques, principally because these insects cannot be reliably followed in the field for observation purposes.

The amplification success of plant DNA from mirids was low (28%), probably because of DNA degradation through extra-oral digestion. Nevertheless, valuable insights into the feeding behaviour of individual bugs could still be made. When using chloroplast sequences for diet analyses a trade-off between amplification success and host plant resolution is evident. Indeed, consensus has not been reached on the best regions to use as a plant DNA barcode, and no single region fits all the requirements [28], [29]. Shorter regions such as the P6 loop of the *trnL* intron provide better amplification success from degraded DNA but lower resolution of host species [23]. As recommended for the broader plant bar-coding effort, diet analyses would most likely benefit from the use of more than one region to balance this trade-off.

Future studies of insects recorded from multiple plant species should evaluate their feeding on ‘incidental hosts’, ones that have no records of juveniles, or from which few insects have been collected. If no evidence of feeding is found then a scientific basis for the removal of such species from host plant lists can be made. Not only can incidence records be refined in this way to represent the ecology of the herbivore more realistically but, conversely, insect feeding on hosts where no observations of insect presence have been made can be detected when an insect collected on a specific plant has indeed fed on another one recently. This is important for applied entomological research, not only in cases such as biocontrol, where the accurate establishment of host plant relationships in the field is critical (e.g. [30]), but also in the study of agricultural pests. An insect that is sampled from a particular crop may have fed on another crop or non crop host plant prior to moving onto the crop in question, and this approach provides a means to recognise this aspect of individual insect behaviour.

Evolutionary studies have increasingly used insect herbivores as systems to investigate speciation [31]–[33], and cases are often portrayed as incipient or ongoing speciation events driven by ongoing selection across two alternative host plant species. There is, however, an alternative explanation for many of these patterns. Speciation may well have occurred in geographically separate populations and, under natural conditions now, host use is differentiated across the two species and gene flow is effectively zero. Evaluating such examples requires, first, that variation in host plant use can be attributed to the individual, the population or the species, and, second, where differences in host use are observed between populations, that contemporary levels of gene flow between these populations is quantified accurately. Both aspects must be evaluated under field conditions because both feeding and mating trials in the laboratory often give equivocal results, probably because of the unintended removal of long range aspects of host and mate searching mechanisms [34]. Our approach provides a way to evaluate the first of these two factors through the analysis of feeding by individuals in the field and their relative abundance on each host. The second can only be accomplished through the sampling of multiple insects from different hosts in the field, and the quantification of contemporary gene flow using multiple loci, e.g. [35].

We hope that the methodological approach developed here will enable not only a more thorough testing of host plant interactions under field conditions, but also a deeper understanding of the evolutionary processes pertaining to insect – host plant relationships.

## Materials and Methods

Host records were collated from the available literature on *C. dilutus* [3]–[6]. Field surveys of host plants and *C. dilutus* abundance were conducted during July and August 2007 in the eastern cropping regions of Australia and the arid interior (Fig. 1). Permits were not required for the collection of this species as it is an economically significant pest, and collections were made at road verges. Sites were dictated by the availability of plants suitable for sampling, which was patchy at best. At each site stands of possible host plants were located for sampling, with each having to consist mostly of one species (>95%), and cover at least 10 m by 10 m. In 6000 km travelled only 22 such sites were located; the remaining terrain was too dry.

*Creontiades dilutus* abundance was quantified using a standardised sweep net sample with an area of 5 m^2^, ten replicates. The adults and juveniles of *C. dilutus* are highly motile, and sweep net sampling has been shown to be a reliable and repeatable method to sample this species [36]. Abundance was recorded, and *C. dilutus* individuals were collected and stored in 96% ethanol for subsequent DNA analysis (up to a maximum of 50). Herbarium specimens of each putative host were collected for identification, and leaf tissue was collected and stored in silica gel for DNA analysis. Herbarium samples were identified using the public reference centre of the Queensland Herbarium (Department of Environment and Resource Management, Brisbane). Putative host plants recorded from the survey in this study were integrated into the list of host plants so far reported in the literature (Table S1). This list was then reduced to those records that specified nymphal bugs had been recorded on the plant in question, and the number of host plant species in each family was plotted (Fig. 2).

Plants in the genus *Cullen* had the highest relative abundance of green mirids (Table S2), but the abundance of an insect on a host plant is also affected by site-specific factors. We therefore analysed *C. dilutus* abundance on a site by site basis, considering only sites where *Cullen* hosts were sampled and more than two *C. dilutus* individuals had been sampled on another host using the standardised sampling outlined above. The abundance of green mirids across different host plants at each of these seven sites (of 22 sites in total) was compared using a one-way ANOVA. Appropriate transformations were applied to the data to conform to ANOVA assumptions (Fig. 3). *Post hoc* pair wise comparisons of means were made using Fisher's LSD test, with the experiment-wise alpha-level (0.05) maintained using a Benjamini-Hochberg correction [37].

To investigate the immediate feeding history of bugs relative to the plant species from which they had been sampled, we amplified chloroplast intergenic spacers from both the insects and plants sampled. We selected sites where sufficient *C. dilutus* had been collected from several hosts including *Cullen*, and we extracted DNA from all plants that had returned at least one mirid in the quantified sampling. DNA was extracted from these putative host plants using a CTAB protocol [38], and from *C. dilutus* thorax and abdomens using QIAGEN DNeasy tissue kits (Qiagen). The *trnL-trnF* intergenic spacer was amplified for both putative hosts, and insect gut contents, using the *trnL* e (B49873: GGTTCAAGTCCCTCTATCCC) and *trnF* f (A50272: ATTTGAACTGGTGACACGAG) primers [14]. PCR was performed using Platinum Taq (Invitrogen), 0.2–0.4 µM of each primer, and 1.5–3 µM of MgCl. PCR cycling conditions were similar to those detailed by [10], with a touchdown of one degree per cycle (18 cycles) from 60°C to 43°C annealing temperature (60 s), and 27 additional cycles at 42°C. Denaturation was 94°C for 30 s, and elongation was 72°C for 45 s. Amplicons were sequenced bi-directionally on an ABI 3730 (Macrogen). Sequences were edited using CodonCode Aligner. Plant derived sequences were used to construct a local BLAST database in Geneious [39], and insect-gut derived sequences were batch blasted (BLASTn) against this database, and against the nr/nt database (NCBI, Genbank). When the BLAST search indicated a hit the insect-derived sequence was pair wise aligned with the plant-derived sequence using ClustalW [40], and a hit was defined using a 2% base difference threshold (Table 1). Host plant sequences (JX134198–JX134221), and gut content sequences (JX134132–JX134197), were deposited in Genbank.

## Supporting Information

Table S1 **Incidence records of the green mirid** ***Creontiades dilutus*** **obtained from a survey of the literature and field survey results from the present study.** (Attached as Excel datafile).
(XLSX)

Table S2 ***Creontiades dilutus*** **collection data showing all sites sampled during the field survey reported in this paper.** (Attached as Excel datafile).
(XLSX)
